# Characteristics and progression of children with acute viral bronchiolitis subjected to mechanical ventilation

**DOI:** 10.5935/0103-507X.20160003

**Published:** 2016

**Authors:** Roberta Ferlini, Flávia Ohlweiler Pinheiro, Cinara Andreolio, Paulo Roberto Antonacci Carvalho, Jefferson Pedro Piva

**Affiliations:** 1 Pediatric Intensive Care Unit, Hospital da Criança Santo Antônio - Porto Alegre (RS), Brazil.; 2 Pediatric Intensive Care Unit, Hospital Moinhos de Vento - Porto Alegre (RS), Brazil.; 3 Pediatric Intensive Care Unit, Hospital de Clínicas de Porto Alegre - Porto Alegre (RS), Brazil.; 4 Department of Pediatrics, Universidade Federal do Rio Grande do Sul - Porto Alegre (RS), Brazil.

**Keywords:** Bronchiolitis, Respiration, artificial, Respiratory syncytial viruses, Edema, Child, Intensive care units

## Abstract

**Objective:**

To analyze the characteristics of children with acute viral bronchiolitis subjected to mechanical ventilation for three consecutive years and to correlate their progression with mechanical ventilation parameters and fluid balance.

**Methods:**

Longitudinal study of a series of infants (< one year old) subjected to mechanical ventilation for acute viral bronchitis from January 2012 to September 2014 in the pediatric intensive care unit. The children's clinical records were reviewed, and their anthropometric data, mechanical ventilation parameters, fluid balance, clinical progression, and major complications were recorded.

**Results:**

Sixty-six infants (3.0 ± 2.0 months old and with an average weight of 4.7 ± 1.4kg) were included, of whom 62% were boys; a virus was identified in 86%. The average duration of mechanical ventilation was 6.5 ± 2.9 days, and the average length of stay in the pediatric intensive care unit was 9.1 ± 3.5 days; the mortality rate was 1.5% (1/66). The peak inspiratory pressure remained at 30cmH_2_O during the first four days of mechanical ventilation and then decreased before extubation (25 cmH_2_O; p < 0.05). Pneumothorax occurred in 10% of the sample and extubation failure in 9%, which was due to upper airway obstruction in half of the cases. The cumulative fluid balance on mechanical ventilation day four was 402 ± 254mL, which corresponds to an increase of 9.0 ± 5.9% in body weight. Thirty-seven patients (56%) exhibited a weight gain of 10% or more, which was not significantly associated with the ventilation parameters on mechanical ventilation day four, extubation failure, duration of mechanical ventilation or length of stay in the pediatric intensive care unit.

**Conclusion:**

The rate of mechanical ventilation for acute viral bronchiolitis remains constant, being associated with low mortality, few adverse effects, and positive cumulative fluid balance during the first days. Better fluid control might reduce the duration of mechanical ventilation.

## Abstract (pt)

**Objetivo:**

Analisar as características de crianças com bronquiolite viral aguda submetidas à ventilação mecânica em 3 anos consecutivos, relacionando a evolução com os parâmetros de ventilação mecânica e o balanço hídrico.

**Métodos:**

Estudo longitudinal de uma série de casos de lactentes (< 1 ano) submetidos à ventilação mecânica por bronquiolite viral aguda entre janeiro de 2012 e setembro de 2014 na unidade de terapia intensiva pediátrica. Os prontuários foram revisados e foram coletados dados antropométricos e dados referentes à ventilação mecânica, ao balanço hídrico, à evolução e a complicações maiores.

**Resultados:**

Incluídos 66 lactentes (3,0 ± 2,0 meses e peso médio de 4,7 ± 1,4kg), sendo 62% do sexo masculino, com etiologia viral identificada em 86%. O tempo médio de ventilação mecânica foi 6,5 ± 2,9 dias, tempo de unidade de terapia intensiva pediátrica de 9,1 ± 3,5 dias, com mortalidade de 1,5% (1/66). O pico de pressão inspiratória médio manteve-se em 30cmH_2_O nos 4 primeiros dias de ventilação mecânica, reduzindo-se na pré-extubação (25cmH_2_O; p < 0,05). Pneumotórax ocorreu em 10% e falha de extubação em 9%, sendo a metade por obstrução alta. O balanço hídrico cumulativo no quarto dia de ventilação mecânica foi 402 ± 254mL, correspondendo a um aumento de 9,0 ± 5,9% no peso. Tiveram aumento de 10% ou mais no peso 37 pacientes (56%), sem associação significativa aos parâmetros ventilatórios no 4º dia de ventilação mecânica, falha de extubação ou tempos de ventilação mecânica e unidade de terapia intensiva pediátrica.

**Conclusão:**

A taxa de ventilação mecânica na bronquiolite viral aguda tem se mantido constante, apresentando baixa mortalidade, poucos efeitos adversos e associada a balanço hídrico cumulativo positivo nos primeiros dias. Melhor controle hídrico poderia reduzir o tempo de ventilação mecânica.

## INTRODUCTION

Acute viral bronchiolitis (AVB) is the most prevalent respiratory disease among children under two years old. Some of the risk factors of AVB are male gender, age under one year old, formula feeding, and low socioeconomic level.^(1,2)^ Approximately 1 to 3% of infants with AVB require hospitalization, and 5 to 15% of them require admission to an intensive care unit (ICU).^(3,4)^ Higher risk of admission to pediatric ICU (PICU), need for mechanical ventilation (MV), long stay at the hospital, and death are associated with low weight, age under 30 days old, prematurity, congenital heart defect, chronic lung disease, and immunodeficiency.^(3,5,6)^ AVB is a seasonal viral disease; the main etiologic agent is respiratory syncytial virus, which is present in more than 50% of the cases.^(7-9)^

AVB characteristically affects the lower airways, being related to bronchiolar obstruction secondary to mucosal edema and the accumulation of mucus and necrotic epithelial cells. AVB is usually a self-limited condition, but in susceptible patients, it might progress to severe respiratory failure requiring MV.^(10)^ Laboratory tests are nonspecific, and the chest radiograph characteristically exhibits pulmonary hyperinflation and interstitial infiltrates to varying degrees.^(11)^

Patients with AVB under MV tend to develop water retention caused by the stimulation of the renin-angiotensin-aldosterone system via the increased secretion of antidiuretic hormone and natriuretic peptide.^(12-14)^ These mechanisms induce sodium retention, with a consequent reduction in urine output. In addition, the use of sedatives and analgesics favors the development of peripheral vasoplegia, with a consequent reduction in the venous return and worsening of peripheral edema.^(15)^

Volume resuscitation is commonly performed in PICUs, being essential in the early management of patients with severe diseases and a high risk of death. Nevertheless, once the acute stage is overcome, attention should be paid to the negative effects of this type of intervention, especially in patients with acute lung disease.^(16)^ Many studies have found an association between positive fluid balance and unfavorable outcomes in critically ill patients.^(17-19)^ A positive cumulative fluid balance over 15% is associated with prolonged MV, a longer ICU stay, and higher mortality.^(19-21)^

The aim of the present study was to analyze the characteristics of children with acute viral bronchiolitis subjected to mechanical ventilation for three consecutive years and to investigate the relationship between the fluid balance with mechanical ventilation parameters and the patients' progression.

## METHODS

The present was a longitudinal study of a case series that included all children under one year old who had AVB and were subjected to MV in the PICU of the *Hospital de Clínicas de Porto Alegre* from January 2012 to September 2014. The study was approved by the research and ethics committee of the *Universidade Federal do Rio Grande do Sul*, with a waiver of informed consent by the children's parents or guardians. The investigators agreed to use the collected data only for the purposes of the study, to communicate the results, and to preserve the participants' anonymity.

In the present study, AVB was defined as a single acute episode of wheezing associated with upper airway symptoms and respiratory dysfunction in infants under one year old and requiring invasive ventilatory support. The chest radiographs of all the infants included in the study exhibited images compatible with obstructive disease (pulmonary hyperinflation with or without interstitial infiltrates). All of the participants were subjected to the detection of viruses in nasopharyngeal secretions by immunofluorescence.

Infants with a history of more than two episodes of AVB; infants with chronic pulmonary disease, recurrent wheezing, previous use of MV due to lung disease, tracheostomy, diagnosis of kidney failure, or congenital heart defects with increased pulmonary blood flow; and very-low-birth-weight premature infants (birth weight less than 1,500g) were excluded from the study.

Data collection was performed by one of the researchers through a review of online medical records stored in the AGHW system, available at the *Hospital de Clínicas de Porto Alegre*, for later analysis of the following data: date of birth, date of admission, date of onset and end of MV, and date of discharge/death; gender; age; weight; Pediatric Index of Mortality 2 (PIM 2) score; comorbidities (prematurity, suspected or confirmed genetic disorders, heart disease with hemodynamic repercussion on echocardiogram assessment and/or coinfection upon admission); viruses detected in nasopharyngeal secretions (adenovirus, parainfluenza virus, influenza B virus, respiratory syncytial virus) and H1N1; complications (coinfection, pneumothorax, acute respiratory distress syndrome, shock, cardiopulmonary arrest); extubation failure within the first 72 hours requiring reintubation; duration of MV, length of stay in the PICU; and time to death.

The MV parameters peak inspiratory pressure (PIP), positive end-expiratory pressure (PEEP), respiratory rate (RR), and fraction of inspired oxygen (FiO_2_) were sequentially recorded after they were adjusted following the first blood gas test after intubation on MV days one, two, three and four, always at midnight. The MV parameters were also recorded before extubation. We defined this schedule to ensure that the data collection would be complete because in the investigated service, the daily patient data are entered into the system at midnight. In addition, the corresponding data are most coherent because the ventilatory status is more stable at the indicated times, as well as a function of the overall management of the investigated PICU. Our service uses SERVOi devices, and the patients were subjected to synchronized intermittent mandatory ventilation (SIMV) with pressure support.

Finally, we also recorded the cumulative fluid balance, in mL/kg/hour, corresponding to the first three days the patients were fully under MV. The fluid balance is calculated by adding the total amount of fluids administered to the patient (per oral and parenteral routes) and then subtracting the fluids lost through physiological excretions, sample collection, and external drains. The cumulative fluid balance was calculated based on the data recorded on MV days one, two, three, and four, always at midnight.

The primary outcomes of the study were the following: percentage of the cumulative fluid balance on MV day four and total duration of MV. The duration of the observation period was established on the grounds that the peak of the bronchiolitis progression occurs close to the fifth day after its onset. The secondary outcomes were as follows: ventilatory parameters on day four and at the time of extubation, length of the PICU stay, and death.

Based on previous studies conducted in the same geographical area,^(22)^ we estimated that the rate of infants under one year old with AVB and requiring MV was 20 to 25 per year. As the study comprised three winter seasons, assuming that 10% of the candidates would exhibit comorbidities or would meet some other exclusion criterion, the number of infants with AVB subjected to MV eligible for inclusion in the study would range from 55 to 60.

The patients' data were transcribed onto an Excel for Windows (Microsoft Office) spreadsheet designed *ad hoc* for the purposes of the present study. The data were subjected to descriptive statistics, expressed in absolute numbers and percentages, and compared using chi-square or Fisher's exact test. Continuous variables were expressed as the mean and standard deviation and compared using Student's *t*-test or analysis of variance (ANOVA). Continuous variables without a normal distribution were expressed as the median and corresponding interquartile range and were compared using the Mann-Whitney U or Kruskal-Wallis test.

## RESULTS

During the study period (2012-2014), which included three winter seasons, 66 infants with AVB were subjected to MV; this population represented 5.6% of the admissions along the investigated period (66/1,178). The average age of the patients was 3.0 ± 2.0 months old, with an average weight of 4.7 ± 1.4kg; 41 (62%) were boys, and 17 (25%) were premature (Table 1). The average PIM2 score was 6.4 ± 2.9. A virus was identified in 86% of the samples, with respiratory syncytial virus being most commonly found (comprising 89% of the cases with a positive diagnosis). Other detected viruses (concomitant or not with the respiratory syncytial virus) were parainfluenza (7%), influenza B (3.5%), and adenovirus (3.5%). H1N1 virus was isolated in 7% of the cases with a positive diagnosis. No infant was dehydrated upon PICU admission. Eighteen patients (27%) received packed red blood cell transfusions while under MV; the hemoglobin concentration at the time of transfusion was 8.1 ± 0.8g/dL.

**Table 1:** Characteristics of infants with acute viral bronchiolitis subjected to mechanical ventilation

Variable	N = 66
Age (months)	3.0 ± 2.0
Weight (kg)	4.7 ± 1.4
Gender (male)	41 (62)
Etiology (positive CRP)	57 (86)
RSV	51 (89)
Other	6 (10)
PIM	6.4 ± 2.9
PRBC transfusion	18 (27)
Prematurity	17 (25)
Pneumothorax	7 (10)
Cardiorespiratory arrest	3 (4.5)
Mortality	1 (1.5)
Length of PICU stay (days)	9.1 ± 3.5
MV duration (days)	6.5 ± 2.9
Extubation failure	6 (9)
Upper airway obstruction	3/6
MV parameters on admission	
PIP (cmH_2_O)	32.1 ± 2.8
PEEP (cmH_2_O)	5.4 ± 0.7
RR (bpm)	20.3 ± 1.7
FiO_2_	0.4 ± 0.1

CRP - C-reactive protein; RSV - respiratory syncytial virus; PIM - Pediatric Index of Mortality; PRBC - packed red blood cells; PICU - pediatric intensive care unit; MV - mechanical ventilation; PIP - peak inspiratory pressure; PEEP - positive end-expiratory pressure; RR - respiratory rate; FiO_2_ - fraction of inspired oxygen. Results are expressed as N (%) or as the mean ± standard deviation.

The average duration of MV was 6.5 ± 2.9 days, and the average length of stay in the PICU was 9.1 ± 3.5 days (Table 1). The mortality rate was 1.5% (1/66); the single case of death occurred on MV day three and was due to refractory septic shock. The rate of extubation failure within the first 48 hours after MV discontinuation was 9% (6/66), due to upper airway obstruction in 50% of the cases and to fatigue in the remainder of the patients. Pneumothorax was detected in 10% of the patients, and cardiorespiratory arrest occurred in 4.5%.

**Figure 1:**
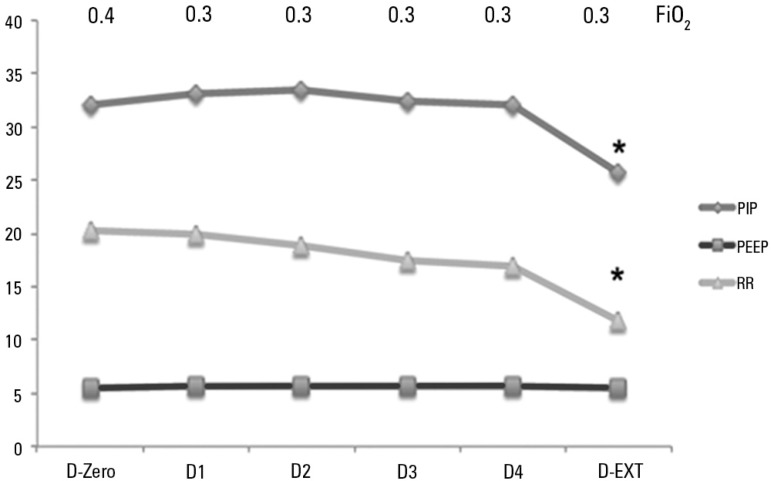
Variation in the fraction of inspired oxygen, peak inspiratory pressure, positive end-expiratory pressure, and respiratory rate corresponding to infants with acute viral bronchiolitis subjected to mechanical ventilation. PIP - peak inspiratory pressure; PEEP - positive end-expiratory pressure; RR - respiratory rate; D - day; D-EXT - extubation day; * p < 0.05.

The average values of the MV parameters after adjustment to the results of the first venous blood gas test were as follows: PIP 32.1 ± 2.8cmH_2_O, PEEP 5.4 ± 0.7cmH_2_O; RR 20.3 ± 1.7bpm (breaths per minute), and FiO_2_ 0.4 ± 0.1. The average PIP remained at approximately 30cmH_2_O during the first four MV days, exhibiting a significant decrease at the pre-extubation assessment only, to approximately 25 cmH_2_O (p < 0.05) with a tidal volume of 10 to 13mL/kg. Moreover, the RR remained stable during the first days under MV (20bpm), decreasing to 10bpm at pre-extubation (p < 0.05) (Figure 1).

**Figure 2:**
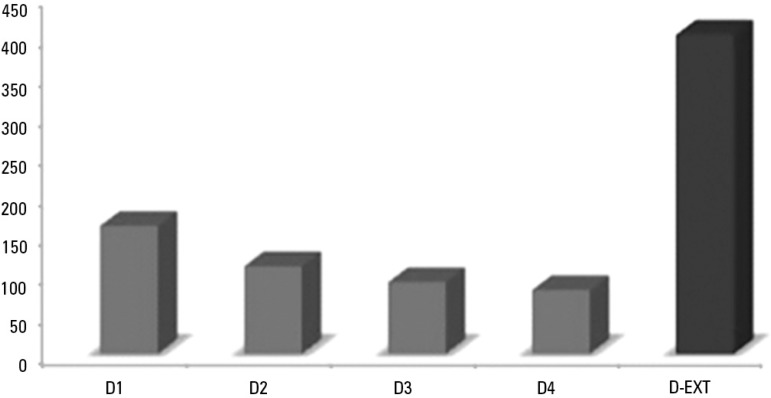
Progression of the daily fluid balance in infants with acute viral bronchiolitis subjected to mechanical ventilation. The average cumulative fluid balance on mechanical ventilation day four was 402 ± 254mL/kg, which corresponds to a variation of 9.0 ± 5.9% in the body weight compared with day one. D - day; D-EXT - extubation day.

The average cumulative fluid balance on MV day four was 402 ± 254mL, which corresponded to an average increase of 9.0 ± 5.9% of the patients' weight on day one. In 29 infants (44%), the body weight increased more than 10% along the first four days of MV. Upon analyzing the sample in two groups, namely, infants with weight variation over or below 10%, we did not find a significant difference in the PIP value on MV day four, MV duration, length of PICU stay, or extubation failure (Table 2 and Figure 2).

**Table 2:** Clinical characteristics of infants with acute viral bronchiolitis subjected to mechanical ventilation according to the degree of water retention on mechanical ventilation day four

Variable	< 10% of weight	> 10% of weight	p-value
	N = 37	N = 29	
Age (months)	3,1 ± 2,0	2,9 ± 2,0	0.6271
Weight (kg)	5,1 ± 1,6	4,1 ± 0,9	0.002
FB1	125 ± 133	205 ± 106	0.009
FB2	77 ± 90	149 ± 132	0.01
Cumulative FB	260 ± 208	582 ± 186	0.0001
Percent weight gain	4.8 ± 3.9	14.3 ± 3.2	0.0001
Length of PICU stay	8.7 ± 3.2	9.7 ± 3.9	0.2507
MV duration	6.4 ± 2.6	6.7 ± 3.3	0.6685
PIP-Dzero	31.7 ± 2.3	34.0 ± 5.7	0.3130
PIP-D4	31.9 ± 1.9	32.5 ± 3.5	0.7238
Extubation failure	4 (11)	2 (7)	0.6789

FB - fluid balance; PICU - pediatric intensive care unit; MV - mechanical ventilation; PIP-Dzero - peak inspiratory pressure on day zero; PIP-D4 - peak inspiratory pressure on day four. Results are expressed as N (%) or as the mean ± standard deviation.

## DISCUSSION

AVB is the most prevalent respiratory illness among children under two years old; its severity is higher among premature infants and infants with previous pulmonary disease, immunodeficiency, or congenital heart defects.^(23)^ In the present study of infants with AVB subjected to MV (conducted along three consecutive winter seasons), we were able to confirm these data, as 25% of the patients were premature and 27% exhibited anemia requiring blood transfusion.

Similar studies performed in various parts of the world have shown that even in the case of severe AVB, high-risk populations (low-weight, small, and premature infants), and prolonged MV (approximately seven days), the mortality rate is low, approximately 1 to 5%.^(22,24)^

According to previous studies, the mortality rate is directly correlated with the respiratory pattern exhibited by the patients, being lower among patients predominantly exhibiting low airway obstruction (classic bronchiolitis). In turn, the rates of complications and mortality are higher among patients who progress to acute respiratory distress syndrome.^(22)^ Notably, the single death in our study was due to refractory septic shock.

By affecting the small airways, AVB promotes an increase in airflow resistance during both inspiration and expiration.^(22,25)^ Bronchiolar inflammation might lead to early closure of the lower airways, with consequent air trapping and even atelectasis of small areas of the lung. When airflow resistance is considerably increased, the maintenance of alveolar ventilation requires higher inspiratory pressures (with eventual recruitment) as well as longer inspiratory and expiratory times as a function of the elevated time constant. Therefore, as in previous studies, we found that a higher PIP (approximately 30cmH_2_O) and a lower RR (20bpm, on average) were used during the period of MV and significantly reduced at pre-extubation only.^(22)^

As recovery from and reversion of the obstructive condition do not occur uniformly, we chose to use high PIP levels even during the pre-extubation stage. This strategy allows the patient to breathe spontaneously between the cycles with low inspiratory pressure (e.g., pressure support), while the mandatory breaths with higher PIP ventilate the lung areas that are still partially collapsed and thus oppose greater resistance, which would not be adequately 'recruited; by ventilation at low pressure. In the present study, we found that this strategy was associated with a low incidence of extubation failure (9%), as half of these cases were due to upper airway obstruction. In addition, the application of this strategy was not associated with an increased incidence of pneumothorax (10% of the cases).

Obviously, the strategy consisting of MV with a higher PIP combined with low RR, low PEEP, and low FiO_2_ values cannot be applied to patients with progressive hypoxemia and whose condition is compatible with acute respiratory distress syndrome. In such cases, ventilatory measures specific for this condition should be implemented.^(26)^ Patents with AVB who progress to acute respiratory distress syndrome might exhibit excessive inactivation of the endogenous surfactant,^(27)^ and in some cases, the ventilation strategy might be complemented by the administration of an exogenous surfactant.^(28)^

Studies performed in recent years have reported a relationship between early fluid overload and increased morbidity and mortality among children with severe acute pulmonary disease, causing a significant increase in the MV duration, need for oxygen, and length of PICU stay.^(19,21,29,30)^ In our study, we found a significant increase in the cumulative fluid balance during the first four days of MV; however, different from the reports in the literature, we were not able to find a relationship between fluid overload and unfavorable outcomes, such as increased MV duration, longer PICU stay, or higher mortality. We did not find any association between positive fluid balance and the need for a higher PIP on the same day. This deleterious effect of positive fluid balance likely did not manifest in the present study as a function of the sample size, which lacked the statistical power required for such a purpose.

The present study has limitations derived from its retrospective design, the possible loss of some information, and the fact that the therapeutic measures were not strictly standardized. In addition, the investigation was conducted at a single center and thus represents the local experience, which may or may not be extrapolated to other centers. These limitations notwithstanding, almost all of our results agree with those reported in the current literature.

## CONCLUSION

On the one hand, the need for mechanical ventilation in acute viral bronchiolitis has not decreased in recent years but has remained constant; on the other hand, the survival expectancy is optimal even in the most severe cases. Such satisfactory outcomes should be attributed to the improvements made to mechanical ventilation equipment, to cumulative medical experience, and to the adoption of consensual protocols for ventilation, sedation, and early cardiovascular support. However, the frequent occurrence of fluid accumulation, as found in the present investigation and other studies with patients subjected to mechanical ventilation, represents a challenge that should be overcome to thereby reduce the associated morbidity.
